# Association Between Body Weight and Clinical Characteristics of Slipped Capital Femoral Epiphysis in Switzerland: A 21-Year Single-Centre Retrospective Analysis (2005–2025)

**DOI:** 10.3390/jcm15166138

**Published:** 2026-08-07

**Authors:** Audrey Meier, Tobias Krause, Carl Alessandro Starvaggi, Milan Milosevic, Kai Ziebarth

**Affiliations:** 1 Department of Pediatric Surgery, University Children’s Hospital, Inselspital, University of Bern, 3010 Bern, Switzerland; audrey.meier@insel.ch (A.M.); tobiastjalf.krause@insel.ch (T.K.); 2 Graduate School for Health Sciences, University of Bern, 3010 Bern, Switzerland; carl.starvaggi@hopitalvs.ch; 3 Department of Pediatric Surgery, Kantonsspital Aarau, 5001 Aarau, Switzerland; milan.milosevic@ksa.ch; 4 Pediatric Orthopedics and Traumatology, Department of Pediatric Surgery, University Children’s Hospital, Inselspital, University of Bern, 3010 Bern, Switzerland

**Keywords:** slipped capital femoral epiphysis, body mass index, body weight, obesity, overweight, adolescent, retrospective studies

## Abstract

**Background:** Epidemiological patterns vary across populations and treatment centres worldwide, with obesity consistently reported as the main risk factor. The primary aim of this study was to characterise age- and sex-adjusted body mass index (BMI) z-scores in children with slipped capital femoral epiphysis (SCFE) treated in Bern, as well as to investigate their association with clinical characteristics. A secondary aim was to compare the observed characteristics with those reported internationally. **Methods:** This is a single-centre, retrospective cohort study conducted at the Department of Pediatric Surgery, University Children’s Hospital, Inselspital, University of Bern, and included patients under the age of 18 who were treated for SCFE between 2005 and 2025. Demographic, clinical, and anthropometric data were collected for all patients, including sex, age at the time of surgery, slip severity using the Southwick angle, intraoperative slip stability, symptom duration, as classified by Fahey/O’Brien, affected side of the hip, as well as height and weight at the time of surgery. The primary outcome was the age- and sex-adjusted BMI z-score at the time of surgery. Analyses stratified by sex, age, and slip severity were prespecified as secondary analyses. All other subgroup analyses were exploratory and hypothesis-generating. **Results:** The final cohort included 90 males (69.23%) and 40 females (30.77%). BMI data were available for 114 of the 130 patients. Two-thirds of the patients (62.3%) were overweight or obese. The median BMI z-score was 1.43 (interquartile range 0.72–2.19; mean 1.31 ± 1.11) and was significantly higher than the expected reference value of 0 (*p* < 0.001). There was no statistically significant difference in BMI z-scores between females and males (*p* = 0.288). A statistically significant association was observed between age at surgery and BMI z-score. Higher age at surgery was associated with lower BMI z-scores (*p* = 0.007). There was a trend towards lower BMI z-scores with increasing slip severity. However, this difference did not reach statistical significance (*p* = 0.158). In an exploratory, hypothesis-generating analysis, patients with stable slips had significantly higher BMI z-scores than those with unstable slips (*p* = 0.008). This association persisted after adjustment for age and after correction for multiple testing (*p*_adj = 0.032). A further exploratory, hypothesis-generating analysis showed that BMI z-scores also differed significantly across symptom duration, as classified by Fahey/O’Brien (*p* = 0.026). The lowest values were observed in the acute-on-chronic group. However, these differences just missed the threshold for statistical significance after adjustment for age and after correction for multiple testing (*p*_adj = 0.052). **Conclusions:** In Switzerland, children diagnosed with SCFE had a significantly higher BMI compared to the reference population. Our study revealed that two-thirds of the children were classified as overweight or obese, with obesity being approximately ten times more prevalent among this group compared to the overall Swiss paediatric population. A higher relative body weight was found to be independently associated with a younger age at surgery, consistent with obesity accelerating skeletal maturation and advancing the manifestation of SCFE. We identified a trend towards lower BMI z-scores with increasing slip severity; however, this finding did not reach statistical significance. Consequently, elevated body weight should be regarded as a marker of predisposition and earlier disease onset rather than of slip severity. Childhood obesity prevention may be relevant to SCFE prevention in Switzerland.

## 1. Introduction

Slipped capital femoral epiphysis (SCFE) is characterised by the displacement of the proximal femoral epiphysis relative to the metaphysis, along the physeal plate [1,2]. Despite its low incidence of 0.5 to 25 cases per 100,000 children, SCFE remains the most common hip disorder in adolescents [2,3]. The variation in incidence is attributed to geographical, racial, and ethnic differences, which are thought to largely reflect differences in body weight distribution among these groups [1,4].

Although the exact aetiology of SCFE remains unclear, it is considered multifactorial, with obesity representing the most consistently reported risk factor [1,5,6,7]. The majority of affected children are overweight or obese [1,8,9]. Body mass index (BMI), adjusted for age and sex, is commonly used to assess weight status.

Overweight children have been reported to have decreased femoral anteversion [6,9]. In combination with increased body weight, this leads to alterations of the epiphysis, repetitive microtrauma, and increased mechanical stress across the growth plate, which may predispose to the development of SCFE [1,4,5,7,9,10,11].

Children with overweight tend to present at a younger age at diagnosis compared to children with normal weight. This results in a disproportionately high prevalence of overweight or obesity among children with SCFE younger than 10 years of age [12]. This finding is likely related to the accelerated skeletal maturation described in overweight children [13,14]. However, the association between obesity and SCFE appears to be less pronounced in girls than in boys [15].

Overweight also appears to be associated with laterality. Descriptive studies have reported that patients with bilateral SCFE tend to have a higher BMI than those with unilateral involvement. Furthermore, patients who subsequently develop contralateral SCFE have been observed to have a higher BMI than those who remain unilateral [1,11].

In addition to the influence of disease occurrence and age at presentation, obesity may also affect slip severity. Obana et al., who investigated SCFE in normal-weight children, reported that non-obese patients are at higher risk for severe slips [14].

Several European studies have investigated the epidemiology of SCFE, some focusing on BMI [15,16,17,18,19,20]; however, no data from Switzerland are available. Switzerland’s combination of high-income status, universal health insurance, and a declining paediatric overweight prevalence [21], contrary to the trend in most other countries [22,23], makes it unclear whether the established association between SCFE and obesity applies unchanged to this population. Characterising SCFE in Switzerland therefore provides an opportunity to test the generalisability of current epidemiological models.

The primary aim of this study was to characterise age- and sex-adjusted BMI z-scores of children with SCFE treated in Switzerland, as well as to investigate their association with clinical characteristics. A secondary aim was to compare the observed characteristics with those reported internationally.

Based on the available evidence, we hypothesised that BMI z-scores would be significantly higher than in the reference population. We also hypothesised that higher BMI z-scores would be associated with a younger age at surgery. Based on the findings of Obana et al. [14], we expected either no association or an inverse association with slip severity. Analyses of slip stability, symptom duration, as classified by Fahey/O’Brien, and laterality were exploratory and hypothesis-generating.

## 2. Materials and Methods

### 2.1. Study Design

This is a single-centre, retrospective cohort study conducted at the Department of Pediatric Surgery, University Children’s Hospital, Inselspital, University of Bern, and included patients who were treated for SCFE between 2005 and 2025. Data were extracted from surgical case lists from this time period. The study is reported in accordance with the STROBE statement for observational studies [24]. Data collection and evaluation for this study were approved by the Cantonal Ethics Committee (project ID 2026-00506).

### 2.2. Participants

Patients under the age of 18 with a confirmed clinical and radiological diagnosis of SCFE who underwent treatment at our institution between 2005 and 2025 were eligible for inclusion. Patients without informed consent were excluded. No additional exclusion criteria were applied. No formal sample size calculation was performed because of the retrospective design of the study. Each patient dataset was assigned a unique study ID and entered into the REDCap database (Research Electronic Data Capture, Version 16.0.39) hosted at university of Bern without any personal identifying information.

### 2.3. Variables

Demographic, clinical, and anthropometric data were collected for all patients. Demographic parameters included sex and age at the time of surgery. Clinical parameters included slip severity based on the Southwick angle, intraoperative slip stability, symptom duration, as classified by Fahey/O’Brien, and affected side of the hip. Anthropometric data included height and weight at the time of surgery.

SCFE can be classified as mild, moderate, or severe. Based on the Southwick angle, mild is defined as an angle less than 30°, moderate as an angle between 30° and 50°, and severe as an angle greater than 50° [2]. SCFE can also be classified as stable or unstable based on an intraoperative assessment of physeal stability, defined by the presence or absence of visible and demonstrable mobility between the metaphysis and the epiphysis [25]. Symptom duration can be classified as acute (symptom duration less than three weeks), chronic (symptom duration longer than three weeks) or acute-on-chronic, according to Fahey/O’Brien [2]. In terms of bilateral SCFE, the hip with the greater Southwick angle was used in analyses involving slip characteristics.

Body mass index was calculated using the formula BMI = weight (kg)/height^2^ (m^2^). Age- and sex-specific BMI standard deviation scores (z-scores) were derived using the LMS method [26] from the German reference values of Kromeyer-Hauschild [27].

This reference was chosen as the primary reference for three reasons. First, it is the BMI reference in routine paediatric use in German-speaking countries and is based on a large Central European population, reflecting the cultural, geographical and linguistic characteristics more closely than the international WHO (World Health Organisation) 2007 reference, which is derived from older United States survey data [28]. Second, it was available and clinically established throughout the entire study period (2005–2025), whereas the first broadly based national Swiss reference was published only in 2019 and updated in 2025 [29], and has yet to be uniformly implemented in clinical practice. Third, using an internationally recognised reference preserves comparability with the existing SCFE literature, in line with the secondary aim of this study.

Because reference populations differ in the period and population from which they were derived, absolute z-score values can vary systematically between references. To ensure that this choice did not influence our conclusions, all analyses were repeated using the WHO 2007 Growth Reference [28] and the Updated Swiss Growth References 2025 [29].

Weight categories were defined using the cut-offs applied to the Kromeyer-Hauschild reference. Overweight was defined as a BMI between the 90th and 97th percentile, and obesity as a BMI above the 97th percentile. Underweight was defined as a BMI below the 10th percentile [27].

### 2.4. Statistical Analysis

Statistical analyses were performed using IBM SPSS Statistics (IBM Corp., Armonk, NY, USA; version 31.0.1.0). Continuous variables were reported as mean ± standard deviation (SD) and as median and interquartile range (IQR). Categorical variables were presented as counts and percentages. The number of patients contributing to each analysis is reported for every variable because of missing data. The confidence interval (CI) was set at 95%, and a *p*-value of ≤0.05 was considered statistically significant.

The normality of the BMI z-score distribution was assessed using the Shapiro–Wilk and Kolmogorov–Smirnov tests, as well as graphical methods. Since the distribution deviated from normality (Shapiro–Wilk *p* < 0.001), non-parametric methods were used for univariable group comparisons.

The Wilcoxon signed-rank test was used to determine if there were significant differences between the BMI z-scores and the expected reference value of 0 in the normative population. The Mann–Whitney U test was used for comparisons between two groups, and the Kruskal–Wallis test was used for comparisons across more than two groups. The Kruskal–Wallis test was also used to evaluate BMI z-score differences across slip severity categories. Associations between continuous variables were assessed using Spearman’s rank correlation coefficient. Associations between categorical variables were assessed using Pearson’s chi-square test or the Fisher–Freeman–Halton exact test when expected cell counts were less than five.

Multiple linear regression was used to examine the association between BMI z-score and age at surgery, sex, and slip severity (mild as the reference category). An exploratory model additionally included slip stability. Multicollinearity was assessed using variance inflation factors, and model assumptions were evaluated by inspection of residual plots.

Missing data were assessed systematically. Little’s test was used to test whether data were missing completely at random [30]. We compared patients with and without missing values regarding age at surgery, sex, slip severity, symptom duration, and BMI z-score. The primary analyses included only complete cases. As a sensitivity analysis, we used multiple imputation for missing BMI z-scores and slip stability values, based on age at surgery, sex, weight, slip severity, and symptom duration. The multivariable models were refitted separately on each imputed dataset, and the resulting estimates were then combined.

To test whether our results depended on the choice of reference, we repeated all analyses using two alternative BMI z-score references: the WHO 2007 Growth Reference [28] and the Updated Swiss Growth References 2025 [29]. Since BMI-based weight categories depend on the reference standard applied, we additionally used the International Obesity Task Force (IOTF) criteria [31] to enable a direct, methodologically consistent comparison with previously published Swiss prevalence data, which had used the same classification system.

The primary outcome was the age- and sex-adjusted BMI z-score at the time of surgery. Analyses stratified by sex, age, and slip severity were prespecified as secondary analyses. All other subgroup analyses were exploratory and hypothesis-generating. Exploratory analyses were corrected for multiple testing using the Benjamini–Hochberg procedure, controlling the false discovery rate at 5% [32].

## 3. Results

**Figure 1:**
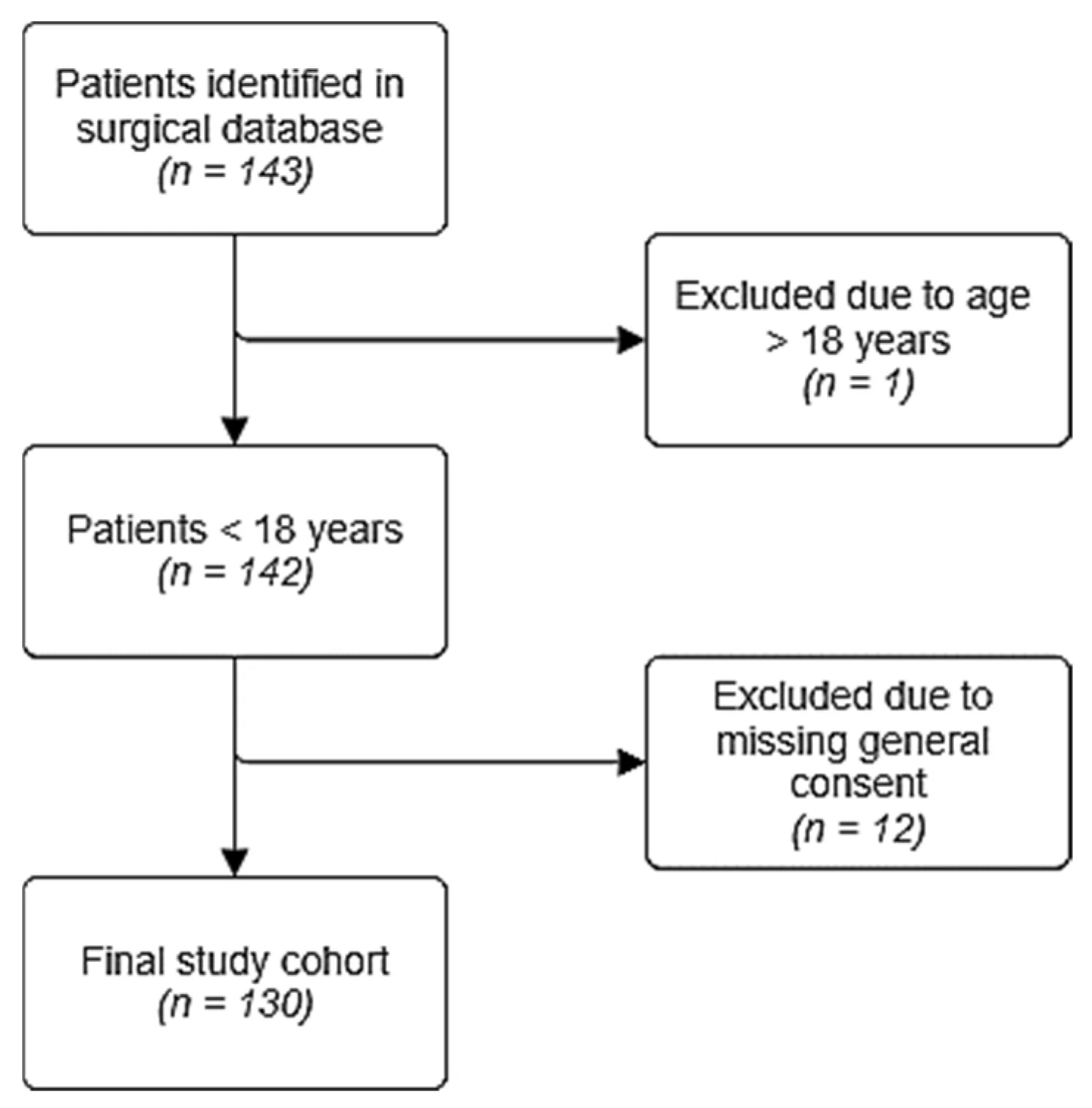
Flow diagram of patient screening, exclusion, and final cohort selection.

We identified 143 patients with confirmed clinical and radiological diagnosis of SCFE. One patient was excluded due to being over 18 years of age. Of the remaining 142 patients, 12 did not provide general consent, resulting in a final cohort of 130 patients (Figure 1).

Due to the retrospective design of the study, missing data were present for several variables. These missing data resulted from incomplete clinical records or radiographic documentation. Sixteen patients (12.31%) had missing height information at the time of surgery, which prevented BMI calculation. However, data from these patients could still be used for statistical analyses of secondary parameters.

The final cohort included 90 males (69.23%) and 40 females (30.77%). BMI data were available for 114 of the 130 patients. Of those patients, three (2.63%) were underweight, 40 (35.09%) had normal weight, 33 (28.95%) were overweight, and 38 (33.33%) were obese.

Data on slip severity were available for all patients, including 37 mild slips (28.46%), 22 moderate slips (16.92%), and 71 severe slips (54.62%). Data on slip stability were available for 83 of the 130 patients, of whom 59 (71.08%) were classified as stable and 24 (28.92%) as unstable. Data on symptom duration, as classified by Fahey/O’Brien, were available for 128 of the 130 patients and showed 12 acute slips (9.38%), 60 chronic slips (46.88%), and 56 acute-on-chronic slips (43.75%). Data on the affected side of the hip were available for all patients. SCFE affected the left hip in 69 patients (53.08%), the right hip in 49 patients (37.69%) and both hips in 12 patients (9.23%) (Table 1).

**Figure 2:**
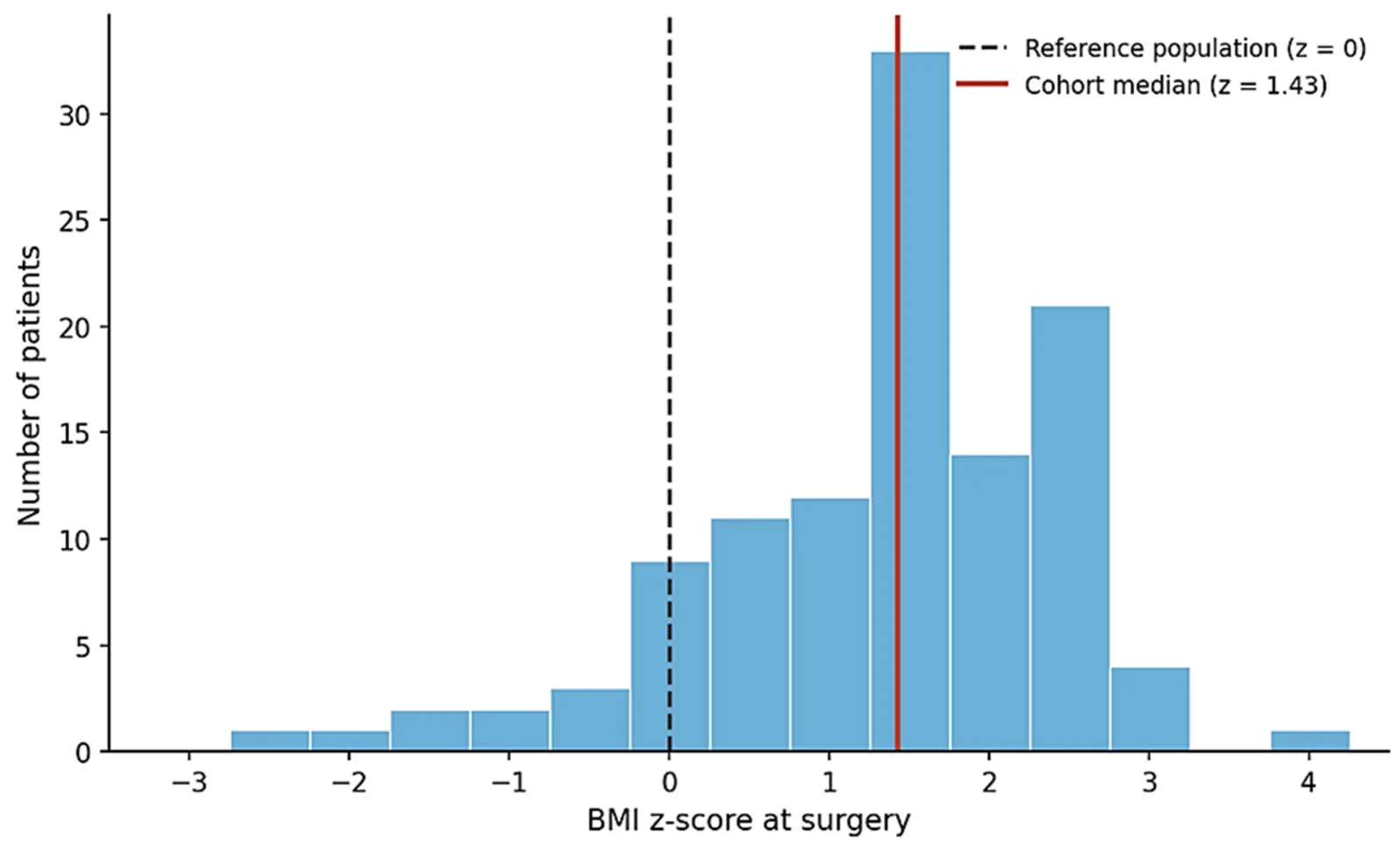
Distribution of age- and sex-adjusted BMI z-score at surgery (*n* = 114). The dashed reference line at z = 0 indicates the expected median of the Kromeyer-Hauschild reference population.

The median age- and sex-adjusted BMI z-score was 1.43 (IQR 0.72–2.19; mean 1.31 ± 1.11) and was significantly higher than the expected reference value of 0 (*p* < 0.001) (Figure 2). Applying identical IOTF criteria, overweight including obesity was present in 68.4% of our cohort versus 13.6% of Swiss children, and obesity in 29.8% versus 2.9% (Table S1) [29].

A statistically significant association was observed between age at surgery and BMI z-score. Higher age at surgery was associated with lower BMI z-scores (*p* = 0.007) (Table 2).

There was no statistically significant difference in BMI z-scores between females and males (*p* = 0.288). There was a trend towards lower BMI z-scores with increasing slip severity; however, this difference did not reach statistical significance (*p* = 0.158) (Table 3).

In an exploratory, hypothesis-generating analysis limited to 74 of the 130 patients with both documented slip stability and available BMI data, patients with stable slips had significantly higher BMI z-scores than those with unstable slips (*p* = 0.008).

A further exploratory, hypothesis-generating analysis of 128 of the 130 patients revealed that BMI z-scores differed significantly across symptom duration, as classified by Fahey/O’Brien (*p* = 0.026). The lowest values were observed in the acute-on-chronic group (Table 4).

The association between slip stability and BMI z-scores persisted after adjustment for age and after correction for multiple testing (*p*_adj = 0.032). The association between symptom duration and BMI z-scores, however, just missed the threshold for statistical significance after adjustment for age and after correction for multiple testing (*p*_adj = 0.052) (Table S2).

Slip severity and symptom duration, as classified by Fahey/O’Brien, were not independent (*p* < 0.001). Severe slips predominantly occurred in the acute-on-chronic group, while mild slips predominantly occurred in the chronic group.

In a multivariable linear regression model with BMI z-score as the dependent variable, younger age was the only independent significant predictor of higher BMI z-scores (*p* = 0.017). Sex (*p* = 0.767) and slip severity (moderate *p* = 0.433; severe *p* = 0.078) were not independently associated (Table 5).

In an exploratory model including slip stability, a younger age at surgery (*p* = 0.002) and an unstable slip (*p* = 0.013) were independently associated with a lower BMI z-score (Table S3).

Missing data were examined systematically. Little’s test did not provide evidence against the assumption of data missing completely at random (*p* = 0.071). Height was missing in 16 of 130 patients and was unrelated to age (*p* = 0.997), sex (*p* = 1.000) and slip severity (*p* = 0.333), but was unevenly distributed across symptom duration, as classified by Fahey/O’Brien (*p* = 0.007), missing in 23.2% of acute-on-chronic vs. 5.0% of chronic presentations. Slip stability was missing in 47 of 130 patients and was unrelated to BMI z-score (*p* = 0.152), sex (*p* = 0.113), slip severity (*p* = 1.000) and symptom duration, as classified by Fahey/O’Brien (*p* = 0.306). However, patients with missing slip stability data were younger (*p* = 0.002) (Table S4).

In a multiple imputation sensitivity analysis using all 130 patients, both independent associations observed in the exploratory model were confirmed. A younger age at surgery (*p* = 0.010) and an unstable slip (*p* = 0.010) remained independently associated with a lower BMI z-score (Table S5).

Twelve patients (9.2%) presented with simultaneous bilateral SCFE and one developed contralateral involvement during follow-up. BMI z-scores were numerically higher in patients with bilateral than with unilateral disease (median 1.69 vs. 1.42; mean 1.75 ± 1.10 vs. 1.27 ± 1.11), but this difference was not statistically significant (*p* = 0.227).

In sensitivity analyses, all analyses were repeated using the WHO 2007 Growth Reference and the Updated Swiss Growth References 2025, in addition to the Kromeyer-Hauschild Reference used in the primary analysis.

Median BMI z-scores differed systematically across references (WHO Reference 1.72, Kromeyer-Hauschild Reference 1.43, Swiss Reference 1.31), reflecting the different periods in which the underlying reference populations were measured. However, all analyses were concordant in direction and in statistical significance. BMI z-scores remained significantly elevated (all *p* < 0.001), did not differ by sex (*p* = 0.264–0.585) or slip severity (*p* = 0.127–0.158), remained significantly higher in stable than in unstable slips (*p* = 0.008–0.010), differed across symptom duration, as classified by Fahey/O’Brien (*p* = 0.026–0.031), and remained inversely associated with age at surgery (all *p* ≤ 0.007) (Table S6).

## 4. Discussion

The recent literature from other countries indicates that most patients who develop SCFE are overweight or obese [1,8,9]. We hypothesised that children with SCFE in Switzerland would also demonstrate elevated age- and sex-adjusted BMI z-scores compared with the reference population. This study’s results support this hypothesis, as two-thirds of the patients were overweight or obese. Applying identical IOTF criteria, our cohort showed an approximately five times higher prevalence of overweight including obesity, and an approximately ten times higher prevalence of obesity alone, compared with Swiss children of the same age [29]. This large discrepancy is the central finding of our study.

Although absolute z-scores differed across references, all analyses were concordant in direction and in statistical significance. The choice of reference therefore affects the absolute magnitude of the z-scores but not their interpretation.

While Obana et al. described a significantly lower BMI in females than in males [14], we found no statistically significant difference in BMI z-scores between females and males. These comparisons should be interpreted with caution because Obana et al. [14] investigated a two-centre cohort from the United States with substantial regional variation in obesity prevalence and classified weight status using the Centers for Disease Control and Prevention growth charts [33], whereas Kromeyer-Hauschild percentiles were used in the present study. These differences in study population and BMI classification may partly explain the discrepant sex-related findings.

Obana et al. also identified a trend towards non-obese patients presenting with a higher Southwick angle [14]. In our cohort, there was also a trend towards lower BMI z-scores with increasing slip severity. However, this difference did not reach statistical significance. These findings further underline the idea that SCFE is a complex condition, with multiple risk factors contributing to disease development.

Furthermore, a statistically significant association was observed between age at surgery and BMI z-score. A higher BMI z-score was independently associated with a younger age at surgery.

Metabolic theories suggest that obesity-related hormonal shifts, such as an earlier pubertal onset and the resulting delay in epiphyseal plate closure, play an important role in the development of SCFE. Furthermore, obesity has been linked to structural changes within the epiphyseal plate itself, including alterations in the shape, size, and organisation of physeal chondrocytes [4]. Biomechanically, obesity is associated with reduced femoral anteversion and increased physeal obliquity, both of which increase the shear forces acting across the growth plate [6,9]. The combination predicts earlier failure.

Consistent with these theories, all four patients younger than 10 years for whom BMI could be calculated were obese. This finding is consistent with a study by Chatziravdeli et al., which reported that more than half of patients across all age groups were overweight, though this was particularly pronounced in younger patients. In patients younger than 10 years, obesity was reported to be especially common [12].

Exploratory analyses revealed that patients with stable slips had significantly higher BMI z-scores than those with unstable slips, as previously demonstrated by Obana et al. [14]. This association persisted after adjustment for age, sex and slip severity, after correction for multiple testing, and in a multiple imputation analysis using all 130 patients. It was also reproduced under all three growth references.

A mechanical model offers a plausible explanation. Stable and unstable slips may reflect two different failure mechanisms of the proximal femoral physis. In stable slips, chronic overload causes repetitive microtrauma, and the physis fails gradually while callus formation and remodelling occur alongside this slow displacement. Unstable slips, in contrast, result from a single acute event in which shear forces exceed the physis’s strength. However, this interpretation remains hypothesis-generating, as it is based on only 74 patients with both documented slip stability and available BMI data.

Taken together, these findings suggest that body weight may be associated with susceptibility to physeal failure and the age at disease onset rather than with the severity of displacement once failure has occurred. Given the retrospective, observational design and the exploratory nature of some analyses, these findings should be considered hypothesis-generating.

Further exploratory analyses showed differences in BMI z-scores across symptom duration, as classified by Fahey/O’Brien, with the lowest values in the acute-on-chronic group. This finding should be interpreted with caution for three reasons. First, it did not remain statistically significant after correction for multiple testing. Second, slip severity and symptom duration, as classified by Fahey/O’Brien, were not independent. Third, height data were missing disproportionately in the acute-on-chronic group.

Twelve patients presented with simultaneous bilateral SCFE and one developed contralateral involvement during follow-up. Among the eleven patients with bilateral involvement with available BMI data, five were obese, two were overweight, and four had normal weight. BMI z-scores were numerically higher in patients with bilateral than with unilateral involvement. However, this difference did not reach statistical significance, most likely reflecting limited power. These findings are descriptively consistent with those reported by Aversano et al., who demonstrated that patients with a higher BMI are at a higher risk for a bilateral occurrence, whether it is simultaneous or sequential [11].

This study has several limitations. First, the retrospective, single-centre design carries an inherent risk of selection and information bias. As a tertiary referral centre, our cohort may be enriched with severe slips, which limits the study’s generalisability. Second, several variables, particularly slip stability and height, had missing values. Multiple imputation confirmed the principal findings; however, because the fraction of missing information for slip stability is substantial, these findings should still be considered exploratory. Third, the small number of patients in some subgroups limited the analyses that could be performed and prevented full adjustment for collinear predictors, such as slip severity, slip stability and symptom duration, as classified by Fahey/O’Brien. Consequently, these findings should be considered exploratory and hypothesis-generating. Finally, as this is an observational study, no causal inferences can be drawn regarding the relationship between body weight and SCFE characteristics.

## 5. Conclusions

In Switzerland, children diagnosed with SCFE had a significantly higher BMI compared to the reference population. Our study revealed that two-thirds of the children were classified as overweight or obese, with obesity being approximately ten times more prevalent among this group compared to the overall Swiss paediatric population.

A higher relative body weight was found to be independently associated with a younger age at surgery, and, in exploratory, hypothesis-generating analyses, with a stable rather than an unstable slip. We identified a trend towards lower BMI z-scores with increasing slip severity; however, this finding did not reach statistical significance.

Elevated body weight may be associated with susceptibility to physeal failure and the age at disease onset rather than with the severity of displacement once failure has occurred.

As this was a retrospective, observational, single-centre study, no causal relationship between body weight and SCFE can be established. Nevertheless, given that childhood overweight remains a public health concern in Switzerland [21], and considering the consistent association observed in our study, paediatric obesity prevention may plausibly contribute to the prevention of SCFE. The determinants of slip severity were not resolved by the present dataset and remain an open question for prospective, purpose-designed studies.

Clinicians and paediatricians in Switzerland should be aware that SCFE is strongly associated with elevated body weight in this population. Elevated body weight should be regarded as a marker of predisposition and earlier disease onset rather than of slip severity.

## Figures and Tables

**Table 1:** Overview of the demographic and clinical characteristics of the study cohort. Denominators (*n*) differ across variables because of missing data. Percentages are based on the available denominator for each variable.

Measures	Counts (%)
Sex (*n* = 130)	
Males	90 (69.23)
Females	40 (30.77)
Weight categories (*n* = 114)	
Obese	38 (33.33)
Overweight	33 (28.95)
Normal weight	40 (35.09)
Underweight	3 (2.63)
Slip severity (*n* = 130)	
Mild	37 (28.46)
Moderate	22 (16.92)
Severe	71 (54.62)
Slip stability (*n* = 83)	
Stable	59 (71.08)
Unstable	24 (28.92)
Fahey/O’Brien (*n* = 128)	
Acute	12 (9.38)
Chronic	60 (46.88)
Acute-on-chronic	56 (43.75)
Affected side of the hip (*n* = 130)	
Left	69 (53.08)
Right	49 (37.69)
Bilateral	12 (9.23)

**Table 2:** Secondary analyses with age stratified by weight categories. Group sizes (*n*) differ across variables because of missing data.

Age [Years]	Mean ± SD	Median (IQR)
Total (*n* = 130)	12.68 ± 1.80	12.60 (11.38–13.95)
Age by weight category		
Obese (*n* = 38)	12.02 ± 1.78	11.91 (11.02–12.84)
Overweight (*n* = 33)	13.04 ± 1.82	13.23 (11.48–14.20)
Normal weight (*n* = 40)	12.90 ± 1.60	13.09 (11.46–13.94)
Underweight (*n* = 3)	13.62 ± 2.44	12.81 (12.25–14.58)

**Table 3:** Secondary analyses of BMI z-scores stratified by sex and slip severity. Group sizes (*n*) differ across variables because of missing data.

Measures	BMI z-Score, Mean ± SD	BMI z-Score, Median (IQR)
Sex		
Males (*n* = 79)	1.25 ± 1.06	1.42 (0.74–1.98)
Females (*n* = 35)	1.46 ± 1.23	1.48 (0.71–2.39)
Slip severity		
Mild (*n* = 35)	1.59 ± 0.90	1.61 (1.28–2.32)
Moderate (*n* = 19)	1.33 ± 1.06	1.48 (0.64–2.22)
Severe (*n* = 60)	1.14 ± 1.22	1.34 (0.45–1.94)

**Table 4:** Exploratory analyses of BMI z-scores stratified by slip stability and symptom duration. Group sizes (*n*) differ across variables because of missing data.

Measures	BMI z-Score, Mean ± SD	BMI z-Score, Median (IQR)
Slip stability		
Stable (*n* = 53)	1.45 ± 1.09	1.42 (0.92–2.28)
Unstable (*n* = 21)	0.66 ± 1.10	0.80 (0.29–1.49)
Fahey/O’Brien		
Acute (*n* = 12)	1.65 ± 0.94	1.45 (1.25–2.50)
Chronic (*n* = 57)	1.53 ± 0.94	1.55 (1.21–2.26)
Acute-on-chronic (*n* = 43)	0.90 ± 1.27	0.93 (0.19–1.88)

**Table 5:** Multivariable linear regression.

Predictor	B	95% CI	*p*
Constant	3.479	1.964 to 4.993	<0.001
Age at surgery (years)	−0.155	−0.282 to −0.028	0.017
Sex (male vs. female)	0.073	−0.415 to 0.562	0.767
Severity (moderate vs. mild)	−0.243	−0.856 to 0.369	0.433
Severity (severe vs. mild)	−0.411	−0.869 to 0.046	0.078

## Data Availability

The data underlying this study are not publicly available due to patient privacy and institutional data protection requirements. De-identified data may be made available from the corresponding author upon reasonable request and subject to institutional and ethical approval.

## Supplementary Materials

The following supporting information can be downloaded at: https://www.mdpi.com/article/10.3390/jcm15166138/s1, Table S1. Comparison with the Swiss paediatric population (IOTF criteria). Table S2. Benjamini-Hochberg correction for multiple testing. Table S3. Exploratory model including slip stability. Table S4. Analyses of missing data. Table S5. Multiple imputation sensitivity analyses including slip stability. Table S6. Sensitivity analyses using different BMI growth references.

## Abbreviations

The following abbreviations are used in this manuscript:

SCFE	Slipped Capital Femoral Epiphysis
BMI	Body Mass Index
CI	Confidence interval
SD	Standard deviation
IQR	Interquartile range
IOTF	International Obesity Task Force
