# Supplementary material for: Association Between Body Weight and Clinical Characteristics of Slipped Capital Femoral Epiphysis in Switzerland: A 21-Year Single-Centre Retrospective Analysis (2005–2025)
Source: J Clin Med. 2026 Aug 7;15(16):6138. doi: 10.3390/jcm15166138 (PMC13513932; doi:10.3390/jcm15166138)
Supplement: Supplementary file 1 [file jcm-15-06138-s001.zip › jcm-4432351-supplementary.pdf]

Table S1. Comparison with the Swiss paediatric population (IOTF criteria).

	Our cohort (n = 114)	Swiss children (n = 19,900)	Ratio
Overweight including obesity	68.4%	13.6%	5.0×
Obesity	29.8%	2.9%	10.3×

Table S2. Benjamini-Hochberg correction for multiple testing.

Rank i	Comparison	*p* (raw)	*p* × m / i	*p* (adjusted)	Significant
1	Slip stability	0.008	0.032	0.032	Yes
2	Fahey/O'Brien category	0.026	0.052	0.052	No
3	Slip severity	0.158	0.211	0.211	No
4	Sex	0.288	0.288	0.288	No

Table S3. Exploratory model including slip stability.

Predictor	B	95% CI	*p*	VIF
Constant	5.402	3.363 to 7.441	<0.001	—
Age at surgery (years)	−0.248	−0.403 to −0.094	0.002	1.17
Sex (male vs. female)	0.195	−0.342 to 0.731	0.472	1.16
Severity: moderate vs. mild	−0.415	−1.123 to 0.292	0.246	1.34
Severity: severe vs. mild	−0.319	−0.930 to 0.293	0.302	1.63
Stability: unstable vs. stable	−0.773	−1.376 to −0.170	0.013	1.29

Table S4. Analyses of missing data.

Missing variable	Compared with	Test	*p*
Height / BMI *(n = 16)*	Age at surgery	Mann-Whitney U	0.997
	Sex	Fisher's exact	1.000
	Slip severity	Fisher-Freeman-Halton	0.333
	Fahey/O'Brien category	Fisher-Freeman-Halton	0.007
Slip stability *(n = 47)*	BMI z-score	Mann-Whitney U	0.152
	Age at surgery	Mann-Whitney U	0.002
	Sex	Fisher's exact	0.113
	Slip severity	Fisher-Freeman-Halton	1.000
	Fahey/O'Brien category	Fisher-Freeman-Halton	0.306

Table S5. Multiple imputation sensitivity analyses including slip stability.

Predictor	B	95% CI	*p*	FMI
Age at surgery (years)	−0.161	−0.283 to −0.039	0.010	0.09
Sex (male vs. female)	0.027	−0.436 to 0.490	0.910	0.10
Severity: moderate vs. mild	−0.190	−0.782 to 0.401	0.528	0.07
Severity: severe vs. mild	−0.185	−0.658 to 0.289	0.444	0.18
Stability: unstable vs. stable	−0.796	−1.397 to −0.195	0.010	0.50

Table S6. Sensitivity analyses using different BMI growth references.

Reference	Reference population	Median z	vs. 0	Sex	Severity	Stability	Fahey	Age (Spearman)
WHO 2007	US NCHS surveys, 1960s–1970s	1.72	<0.001	0.585	0.127	0.010	0.030	ϱ=−0.31, *p*=0.001
Kromeyer-Hauschild 2001	17 German regional samples, 1985–1999	1.43	<0.001	0.288	0.158	0.008	0.026	ϱ=−0.25, *p*=0.007
Swiss Growth References 2019+2025	43,290 Swiss children, 2012–2025	1.31	<0.001	0.264	0.141	0.010	0.031	ϱ=−0.30, *p*=0.001
